# Validation of the Netherlands physical activity questionnaire in Brazilian children

**DOI:** 10.1186/1479-5868-8-45

**Published:** 2011-05-21

**Authors:** Renata M Bielemann, Felipe F Reichert, Vera MV Paniz, Denise P Gigante

**Affiliations:** 1 Post-Graduate Program in Epidemiology, Federal University of Pelotas, Pelotas, Brazil; 2 Physical Activity Epidemiology Research Group, Federal University of Pelotas, Pelotas, Brazil; 3 Post-Graduate Program in Physical Education, Federal University of Pelotas, Pelotas, Brazil; 4 Post-Graduate Program in Population Health, University of Vale do Rio dos Sinos, São Leopoldo, Brazil

## Abstract

**Background:**

Physical activity instruments can be subjective or objective. There is a need to assess the reliability of these instruments, especially for researches in children. The aim of this study was to determine the validity of the Netherlands Physical Activity Questionnaire (NPAQ).

**Methods:**

Population under study were Brazilian children aged 4 to 11 years old, enrolled in a population-based study. Data collection took place in two distinct moments: 1) application of the NPAQ by face-to-face interviews with mothers' children and 2) utilization of accelerometers by children as the reference method. GT1M Actigraph accelerometer was worn for five consecutive days. Validity analyses were performed by sensitivity and specificity and ROC (Receiver Operator Characteristic) curve.

**Results:**

Two hundred and thirty nine children participated in both phases of the study. A total of 73.2% children achieved the recommendation of 60 min/day of moderate to vigorous physical activity. The mean and median of the NPAQ score were 25.5 and 26, respectively. The score ranged from 7 to 35 points. The correlation coefficient between the NPAQ and the time spent in moderate to vigorous physical activities was 0.27. Based on the area under the ROC curve, the median value presented the best indicators of sensitivity (59.4%) and specificity (60.9%), and the area under curve was 0.63. The predictive capacity of the NPAQ to identify active children was high regardless the cut-off point chosen. This capacity was even higher if the score was higher than 30.

**Conclusions:**

Based on sensitivity and specificity values, the NPAQ did not show satisfactory validity. The comparison of the reliability of the NPAQ with other instruments is limited, but correlation coefficients found in this study are similar to others. Physical activity level of children estimated from the NPAQ must be interpreted cautiously, and objective measures such as accelerometers should be encouraged.

## Background

Childhood physical activity may be beneficial for the life course [1,2]. Physical activity practice plays a role on the body weight during childhood and in the life cycle including the impact on the obesity-related diseases [1,3]. Thus, the use of reliable approaches to estimate childhood physical activity is warranted.

Physical activity can be estimated either objectively or subjectively. Accelerometers are objective instruments that have been used to estimate physical activity in epidemiological studies [4,5]. These motion sensors have been shown to be effective when compared to subjective methods of physical activity assessment [6]. Questionnaires are subjective methods, feasible, fast and cheap as compared with accelerometer [7]. While logistics and cost aspects impair the use of motion sensors in large samples, subjective instruments are more prone to bias, mainly among children [8].

Due to the inability of children to report their physical activity accurately [8], questionnaires are usually administered to another person, such as a parent or a teacher [6]. Studies on the validity of questionnaires to estimate physical activity in children are available, but usually statistical analyses present weak points [6,9].

The Netherlands Physical Activity Questionnaire (NPAQ) is an instrument in which parents report their children preference for some activities. Some of these activities are closely related to physical activity, such as playing sports, while others address sedentary behaviors such as reading. The score obtained from this questionnaire was originally tested as a numerical variable, indicating the likelihood of a child being in higher or lower categories of physical activity [10]. However, the ability of the questionnaire to discriminate between active and inactive children has not been assessed. This questionnaire was chosen because it contains a small number of questions, it includes usual activities performed by Brazilian children, it is workable in large scale studies and allows the assessment of cut-off points or specific questions that are more likely to categorize children as sufficiently active or not. Thus, the aim of the present study is to identify cut off points of the NPAQ to accurately categorize children as physically active or inactive. We used accelerometry as the gold standard measure of physical activity for comparison.

## Methods

### Study Design and Sampling

A cross-sectional population based sample of children was undertaken in Pelotas, a medium-sized (323,000 inhabitants; 93% living in the urban area) Southern Brazilian city. Pelotas has a Gini index (an income inequality index) and a mean family income similar to the rest of the country.

The sample size calculation was based on sensitivity and specificity values estimated by the Fletcher's equation[11]. The following parameters were considered: a) sensitivity and specificity of 75%; b) acceptable error of 10 percentage points; c) physical activity prevalence of 60% (defined by achieving or not the cut-off point of at least 60 min/day of moderate to physical activities, which is in accordance with current recommendations[12,13]); d) confidence level of 95% and; d) number of children in the population (i.e. 37 thousand children in the urban area of Pelotas according to Brazilian Institute of Geography and Statistics - IBGE). Based on these parameters, 72 *active *children would be necessary - corresponding to 120 children sampled for the sensitivity - and 72 *inactive *children - corresponding to a total of 180 children for the specificity. We added 20% to compensate for refusals and losses. Thus, 216 children were the final sample size estimated.

Children were selected through a multi-stage sampling process. Firstly, all 404 census tracts of the city were sorted according to their mean income. Each census tracts comprises approximately 300 households and information on the income was provided by the Brazilian Institute of Geography and Statistics IBGE. Because this study is part of a larger health survey, and other outcomes required larger samples, a total 130 census tracts were selected with probability proportional to their size. In the next step, all households from the selected census tracts were listed and 10 of them from each tract were selected systematically. All children from 4 to 10 years of age residents in the sampled households were eligible to take part in this study. This age group was chosen because there are few studies with Brazilian preschool and school children. Besides, there is not a valid physical activity questionnaire for this age-group available in Portuguese.

### Questionnaire

Interviews were carried out with mothers from January to May, 2010. When mothers could not be contacted, the questionnaire was responded by another person responsible for looking after the child. In such cases, women related to the child (mother-in-law, grandmother, aunt, sister, etc.) were preferred, and in case of unavailability of any of them, the father was invited to participate.

The Netherlands Physical Activity Questionnaire (NPAQ) was administered to respondents during face-to-face interviews. The questionnaire was translated into Portuguese and has been used in Brazilian children with no evidence of understanding problems. Some activity examples used in the questionnaire were altered to represent usual Brazilian activities, but the original structure of the questionnaire was unchanged [10]. The third question, which refers to the enjoyment of playing sports, had its score reversed. Furthermore, the fifth question, which refers to the enjoyment of reading, was modified to represent enjoyment of reading magazines, drawing or painting. Each of the seven questions had a score of one to five points. The less active option (example: "He always like to play inside the school or the house") socred one and the most active option (example: "He always like to play outside or in the yard") scored five points. The final score was the sum of all scores.

Questions about demographics and socioeconomic characteristics about the respondent and the child were also collected. The variables studied were sex and age of the child, age and schooling of the respondents, and income and socioeconomic status of the child's family. The Brazilian Association of Research Institute (ABEP), which considers household assets, full-time housekeepers, and head-of-family's schooling was used to determine socioeconomic status. ABEP divides families into five categories, from A (wealthiest) to E (poorest) [14].

### Physical activity measured by accelerometry

Children's physical activity was measured by accelerometers (Actigraph GT1M - LLC, Fort Walton Beach, FL, USA) from February to August of 2010. Children were oriented to wear hip accelerometers from Saturday to Wednesday, during 24 h/day, except while bathing or swimming. The epoch was set to 5 s, because longer intervals may not capture important bouts of children activities. Participants were instructed to register in a diary if they did not wear the device for more than 1 hour.

Data from the accelerometers were processed in Actilife 4.4.1 software and analyzed in the MAHUffe (http://www.mrc-epid.cam.ac.uk). The first (Saturday) and last day (Wednesday) were excluded from analyses. Furthermore, those with < 600 min/day of data or >10 min of consecutive zero counts were excluded. The following thresholds were used to classify physical activity intensities: a) sedentary activities - 0 - 100 counts per minute (cpm); b) light activities - 101 to 1999 cpm; c) moderate activities - 2000 to 4999 cpm; d) vigorous activities - ≥5000 cpm. The lower limit for the moderate activities threshold corresponds (in children) to a walking pace of around 3-4 km/h [15]. Only activities of at least moderate intensity and duration of ≥10 min (a buffer of at most 2 min in activities below moderate intensity was allowed) were counted to the score of minutes per day of physical activity.

Variables analyzed from the accelerometer data were total counts, counts per minute, mean time spent in sedentary and moderate to vigorous activities, and sufficient physical activity (yes/no).

### Statistical analyses

Descriptive analyses of demographic and socioeconomic variables are presented. Association between the score of each question of the questionnaire and the prevalence of sufficient physical active (i.e. ≥60 min/day of moderate to vigorous activities) was analyzed by chi-square tests. Pearson correlation coefficients were calculated between the NPAQ results and the accelerometer variables. Sensitivity, specificity and predictive values for different cut-off points of the questionnaire were calculated using data from the accelerometer as a reference method. The ROC (Receiver Operator Characteristic) curve was built from the results of this later analysis.

### Ethical approval

The study was approved by the Ethics Committee of the Medicine School of Federal University of Pelotas.

Written informed consent was obtained from every mother prior to the interviews.

## Results

### Description of the sample

A total of 369 children, out of the 379 located participated in the study (2.6% of non-response rate). The accelerometer was used by 239 children (45 children (12.6%) refused to participate, 48 (13.0%) could not be located and 37 (10.0%) did not provide valid data on the accelerometer). Therefore, the final response rate was 64.8%.

Table 1 shows the demographic and socioeconomic characteristics of the sample. Most of the children were male and almost 30% were aged 8-9 years old. Almost half of the children had an intermediary socioeconomic status and most of the mothers were not employed when the interview took place, were in the 30-39 age group and had 5 to 8 years of formal schooling. Table 1 also shows that the response proportion was lower in children whose mothers were in the extreme categories of age or intermediate categories of schooling than their counterparts.

**Table 1:** Description of the sample in terms of demographic and socioeconomic variables. Pelotas, Brazil, 2010.

Variables	Sample interviewed		Sample interviewed and accelerometry data		p*
			
	N	%	N	%	
**Sex**					0.7
Male	192	52.0	123	64.1	
Female	177	48.0	116	65.5	
**Age**					0.1
4-5 years	103	27.9	59	57.3	
6-7 years	89	24.1	62	69.7	
8-9 years	109	29.5	68	62.4	
10-11 years	68	18.5	50	73.5	
**Maternal age**					0.009
< 30 years	103	28.0	57	55.3	
30-39 years	169	45.9	108	63.9	
≥ 40 years	96	26.1	73	76.0	
**Socioeconomic level**					0.5
A/B (higher)	44	11.9	29	65.9	
C	167	45.3	113	67.7	
D/E	158	42.8	97	61.4	
**Maternal schooling**					0.006
0-4 years	64	17.4	53	82.8	
5-8 years	150	40.7	89	59.3	
9-11 years	107	29.1	64	59.8	
≥12 years	47	12.8	32	68.1	
**Maternal work**					0.9
Yes	171	47.1	110	64.3	
No	192	52.9	125	65.1	

* Chi-square test for comparison between the sample interviewed and with accelerometer data.

The questionnaire score and the numerical variables from accelerometers are presented in Table 2. The range of the score for the NPAQ data included the minimum and maximum possible values of the instrument (seven and 35). The mean and median values were similar and 95% of the sample had a score between 15 and 35. Regarding the accelerometer data, the mean time in moderate to vigorous activities (≥2000 cpm) was slightly above 60 min/day and mean time in sedentary activities was roughly 10 h/day.

**Table 2:** Description of the physical activity variables from the Netherlands Physical Activity Questionnaire (NPAQ) and accelerometer. Pelotas, Brazil, 2010.

Variable	Minimum	Maximum	Median	Mean	Standard Deviation
**NPAQ score (points)**	7	35	26	25.5	4.8
**Daily counts**	124333.3	1881138	450235	469247.3	171237.8
**Mean counts per minute**	141.8	2005.5	492.4	516.6	190.1
**Daily minutes of sedentary activity**	303.8	817.8	623.1	605.6	103.4
**Daily minutes of moderate activity**	14.9	125.5	64.0	64.5	20.8
**Daily minutes of vigorous activity**	1.2	52.4	12.3	13.6	8.4
**Daily minutes of moderate to vigorous activity**	16.3	177. 9	77.2	78.1	27.2

### Comparison between instruments

The following results include data from the 239 children that provided accelerometry data. Pearson correlation coefficients between the NPAQ and accelerometry are shown in table 3. Low correlation coefficients were observed between accelerometer data and the first five questions of the NPAQ. Coefficients were higher for the last two questions, with the last question showing the highest correlation coefficient (r = 0.27). In addition, the last question presented the strongest negative correlation between time spent in sedentary activities and increased score. It should be highlighted that the correlation coefficient between time spent in moderate to vigorous activities with the last question is similar to the coefficient observed for the NPAQ as a whole (r = 0.27).

**Table 3:** Correlation coefficients between each question and score of NPAQ and accelerometry information. Pelotas, Brazil, 2010.

Question	Daily Counts	Mean counts per minute	Daily minutes of sedentary activity	Daily minutes of moderate activity	Daily minutes of vigorous activity	Daily minutes of moderate to vigorous activity
**1**	0.08	0.08	-0.01	0.09	0.05	0.08
**2**	0.12	0.06	0.07	0.12	0.11	0.13
**3**	0.12	0.09	0.00	0.16	0.08	0.15
**4**	0.08	0.11	-0.16	0.07	0.04	0.07
**5**	0.09	0.07	-0.02	0.17	0.13	0.17
**6**	0.21	0.17	0.02	0.19	0.17	0.20
**7**	0.26	0.29	-0.25	0.26	0.24	0.27
**All (1-7)**	0.24	0.21	-0.08	0.27	0.21	0.27

Sufficient physical activity prevalence (i.e. ≥60 min/day of moderate to vigorous activities) was 73.2% (95%CI = 67.6 - 78.9). Table 4 presents the association between sufficient physical activity prevalence and the alternatives of answer of the NPAQ. Children who "always" or "almost always" do not enjoy drawing, painting or reading magazines were 18 percentage points (p = 0.04) more active than those who reported that "always" or "almost always" do enjoy these activities. Children who like to play outside or in the backyard are 20 percentage points more active than those who prefer to play inside the house or at school (p = 0.01). Prevalence of sufficient activity was around 40% higher among those whose mothers claimed they were more active than other children from the same age (p = 0.005).

**Table 4:** Prevalence of sufficient physical activity according to categories of response to the Netherlands Physical Activity Questionnaire

	Always or Almost always (1 or 2 points)		About equal (3 points)		Almost always or Always (4 or 5 points)			p*
			
	N	%	N	%	N	%		
Prefers to play alone	40	70.0	26	65.4	173	75.1	Prefers to play with other children	0.5

Prefers quiet games like puzzle, cards, toys fit	57	68.4	34	61.8	148	77.7	Prefers vigorous game like running, climbing things, jump rope	0.11

Dislikes playing sports	23	69.6	10	70	206	73.8	Likes playing sports like soccer and ride a bicycle	0.9

Is more introverted, quiet, likes to stay home	48	72.9	18	77.8	173	72.8	Is more extroverted, likes to outgoing	0.9

Likes drawing, painting or reading magazines	174	69.5	16	68.8	49	87.8	Dislikes drawing, painting or reading magazines	0.04

Prefers to play inside the house or school	50	58.0	43	69.8	146	79.5	Prefers to play outside or in the yard	0.01

Less physically active compared to other children of same age	32	59.4	84	65.5	123	82.1	More physically active compared to other children of same age	0.005

Pelotas, Brazil, 2010.

* Chi-square test.

### Validity of the NPAQ in Brazilian children

**Figure 1:**
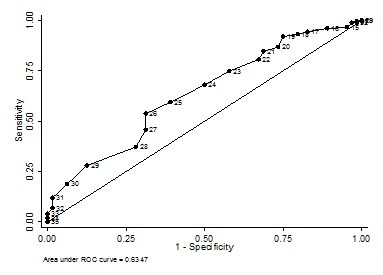
***Receiver Operating Characteristic *(*ROC*) curve for classification of physical activity level from the Netherlands Physical Activity Questionnaire**.

Sensitivity and specificity analyses are presented in Table 5. As expect, an increase in the cut-off point of the NPAQ score was associated to a decrease in sensitivity and increase in specificity. Acceptable values of these two measures of validity were observed to cut-off points around the mean and median of the score. The cut-off point of 25, for example, had a sensitivity of 68.0% (IC_95% _= 60.5 - 74.8) presented a specificity of 50.0% (95%CI = 37.2 - 62.8). It means that for every two inactive children, at least one would be considered active. Using the median of the score (26), the proportions of sensitivity and specificity were around 60%. Figure 1 shows the ROC curve to the values of sensitivity and specificity showed in Table 4. The best cut-off point indicated by the curve is 26. The area under the curve was 0.63 (95%CI = 0.57 - 0.70).

**Table 5:** Sensitivity and specificity of the Netherlands Physical Activity Questionnaire according to different cut-off points.

Cut-off point	**Sensitivity (CI**_**95%**_**)**	**Specificity (CI**_**95%**_**)**
≥ 7	100.0	0.0
≥ 11	99.4 (96.9-100.0)	0.0 (0.0-5.6)
≥ 12	99.4 (96.9-100.0)	1.6 (0.0-8.4)
≥ 13	98.9 (95.9-99.9)	1.56 (0.0-8.4)
≥ 15	98.9 (95.9-99.9)	3.1 (0.4-10.8)
≥ 16	96.6 (92.7-98.7)	4.7 (1.0-13.1)
≥ 17	96.0 (91.9-98.4)	10.9 (4.5-21.2)
≥ 18	94.3 (89.7-97.2)	17.2 (8.9-28.7)
≥ 19	93.1 (88.3-96.4)	20.3 (11.3-32.2)
≥ 20	92.0 (86.9-95.6)	25.0 (15.0-37.4)
≥ 21	86.9 (80.9-91.5)	26.6 (16.3-39.1)
≥ 22	84.6 (78.4-89.6)	31.3 (20.2-44.1)
≥ 23	80.6 (73.9-86.2)	32.8 (21.6-45.7)
≥ 24	74.9 (67.8-81.1)	42.2 (29.9-55.2)
≥ 25	68.0 (60.5-74.8)	50.0 (37.2-62.8)
≥ 26	59.4 (51.8-66.8)	60.9 (47.9-72.9)
≥ 27	53.7 (46.0-61.3)	68.8 (55.9-79.8)
≥ 28	45.7 (38.2-53.4)	68.8 (55.9-79.8)
≥ 29	37.1 (30.0-44.8)	71.9 (59.2-82.4)
≥ 30	28.0 (21.5-35.3)	87.5 (76.8-94.4)
≥ 31	18.9 (13.4-25.5)	93.8 (84.8-98.3)
≥ 32	12.0 (7.6-17.8)	98.4 (91.6-100.0)
≥ 33	6.9 (3.6-11.7)	98.4 (91.6-100.0)
≥ 34	4.0 (1.6-8.1)	100.0 (94.4-100.0)
= 35	1.7 (0.4-4.9)	100.0 (94.4-100.0)

The Table 6 shows the NPAQ positive and negative predictive values. The NPAQ predictive value in relation to accelerometry showed higher predictive values in all the cut-off points evaluated. In contrast, most negative predictive values were below 50%. An increased predictive capacity to the active children was found from the 30 points of the NPAQ.

**Table 6:** Positive and negative predictive values to different cut-off points of the Netherlands Physical Activity Questionnaire

Cut-off point	**Positive predictive value (CI**_**95%**_**)**	**Negative predictive value (CI**_**95%**_**)**
≥ 7		
≥ 11	73.1 (67.0-78.6)	0.0 (0.0-97.5)
≥ 12	73.4 (67.3-78.9)	50.0 (1.3-98.7)
≥ 13	73.3 (67.2-78.8)	33.3 (0.8-90.6)
≥ 15	73.6 (67.5-79.1)	50.0 (6.8-93.2)
≥ 16	73.5 (67.3-79.1)	33.3 (7.5-70.1)
≥ 17	74.7 (68.5-80.2)	50.0 (23.0-77.0)
≥ 18	75.7 (69.4-81.2)	52.4 (29.8-74.3)
≥ 19	76.2 (69.9-81.7)	52.0 (31.3-72.2)
≥ 20	77.0 (70.7-82.6)	53.3 (34.3-71.7)
≥ 21	76.4 (69.9-82.1)	42.5 (27.0-59.1)
≥ 22	77.1 (70.5-82.8)	42.6 (28.3-57.8)
≥ 23	76.6 (69.8-82.5)	38.2 (25.4-52.3)
≥ 24	78.0 (70.9-84.0)	38.0 (26.8-50.3)
≥ 25	78.8 (71.4-85.0)	36.4 (26.4-47.3)
≥ 26	80.6 (72.7-87.0)	35.5 (26.6-45.1)
≥ 27	82.5 (74.2-88.9)	35.2 (26.9-44.2)
≥ 28	80.0 (70.8-87.3)	31.7 (24.0-40.1)
≥ 29	78.3 (67.9-86.6)	29.5 (22.5-37.3)
≥ 30	86.0 (74.2-93.7)	30.8 (24.2-38.0)
≥ 31	89.2 (74.6-97.0)	29.7 (23.5-36.5)
≥ 32	95.5 (77.2-99.9)	29.0 (23.1-35.6)
≥ 33	92.3 (64.0-99.8)	27.9 (22.1-34.2)
≥ 34	100.0 (59.0-100.0)	27.6 (21.9-33.8)
= 35	100.0 (29.2-100.)	27.1 (21.6-33.3)

Pelotas, Brazil, 2010.

## Discussion

The present population based-study verified the validity of a subjective instrument (NPAQ) compared to an objective instrument (accelerometer) to assess physical activity of Brazilian children. The results indicate that the median value of the NPAQ was the most accurate to classify children as sufficient active or inactive. However, sensitivity and specificity values were low and the area under the ROC was small. One should consider that the prevalence of sufficient physical activity estimated by accelerometer was high, thus, the positive predictive value was also high.

Some methodological issues regarding the use of the accelerometer shall be highlighted. Parents' concern about the accelerometers and lack of cooperation of children to wear the devices and might have contributed to the non-response rate. Mean daily time of accelerometer data ranged from 600 to 1200 min, and 50% of children wore it for less than 950 min. Therefore, children did not wear the monitors 24 h/day, as recommended, and physical activity level might be underestimated. Few studies described the mean time of wearing accelerometer. Only two studies that described this analysis (both with adolescents) were located. The first one was carried out in the same city of the current study (Pelotas), and the mean time of registered activities was 921 min/day with a range of 620 to 1266 min [16]. Another study took place in Madrid and the mean time of registered activities was 789 min in the second day of use [17]. Accelerometers are recommended to be worn during at least three days and during each day at least 600 min of activities must be recorded to represent physical activity patterns in children [18]. Other studies addressing validity and prevalence of physical activity have adopted the criteria of 600 min/day of accelerometer data as the minimum to be acceptable [19,20].

The high prevalence of sufficient physical activity found in this study indicate that three every four children achieve the guideline of 60 min/day of moderate to vigorous physical activities. The higher the physical activity level, the lower the adiposity level [19,20], thus, having a physical activity level higher than the current recommendation may, in fact, be desirable.

The choice of a cutoff point to dichotomize the children in active or inactive was necessary to run the validity analyses. The threshold used (60 min/day of moderate to vigorous activities) is in accordance with current recommendations for this age group [12,13]. The need for an instrument to be used in children is explained by the importance to accurately evaluate physical activity in this age group [7], and also because of the diversity of instruments that had their validities inadequately analyzed [9]. In the present study, the NPAQ validity was analyzed by sensitivity and specificity tests, and ROC curve.

The proposal of the NPAQ is to identify patterns of a given set of behaviors, thus, limiting the inherent recall error that arises when an instrument aims to evaluate the exact time and frequency of physical activities [10]. Nonetheless, based on the values of sensitivity and specificity, the results shall be interpreted with caution because of the high probability of misclassification.

According to the authors from the NPAQ, the ability of the questionnaire to distinguish between active and inactive children is mainly observed in the extremes of the score distribution [10]. In the current study, the likelihood of children with high score in the NPAQ to be considered active by accelerometry was high (the prevalence of sufficient physical activity in children in the highest tertile of NPAQ was 86% - data not shown). It should be highlighted that, despite the fact that NPAQ cannot classify children according to the current recommendation (i.e. minutes of moderate to vigorous activities), it clearly shows that the higher the NPAQ score, the higher the prevalence of sufficient physical activity. Thereby, this questionnaire is useful to distinguish groups of children more likely to be inactive.

Some authors consider correlation coefficients from 0.3 to 0.5 to be an indicative that the instrument is valid [21]. Thus, the correlation coefficients found in the present study are in accordance with other studies, but it indicates only weak associations [22-24]. This analysis showed that the last question of the NPAQ had the highest correlation coefficient. This question asks the mother to compare her child's physical activity level to other children of the same age. Similar results based on this question were observed in adolescents [25].

As the use of questionnaires to estimate children's physical activity level has important limitations, the use of objective measures must be encouraged, mainly in low and middle income countries. Although physical activity is an unstable behavior, accelerometers are valid instruments. In addition, the assessment of physical fitness, which is more stable than physical activity, could also be evaluated in studies that aim to determine the etiological association between physical activity and diseases [26].

## Conclusions

The NPAQ showed poor sensitivity and specificity in Brazilian children aged 4 to 11 years old. However, the questionnaire has a good predictive value when used in populations with high prevalence of sufficient physical activity. Moreover, the values of the correlation coefficients were similar to those found in other questionnaires assessing physical activity. The questionnaire may be useful to classify individuals into active or inactive. This is important especially in pediatric populations, which performs specific activities and motivations differ from other age groups.

## Competing interests

The authors declare that they have no competing interests.

## Authors' contributions

RB conceptualized the study, coordinated field work, run the analyses and wrote the manuscript. FR and VR contributed to the writing and revision of the manuscript. DG conceptualized the study, contributed to the writing and revision of the manuscript. All authors read and approved the final version of the manuscript.
